# The Relationship between Reactive Oxygen Species and Cardiac Fibrosis in the Dahl Salt-Sensitive Rat under ACEI Administration

**DOI:** 10.1155/2012/105316

**Published:** 2012-03-05

**Authors:** Ryou Tanaka, Miki Shimizu

**Affiliations:** Department of Veterinary Surgery, Faculty of Veterinary Medicine, Tokyo University of Agriculture and Technology, 3-5-8 Saiwai-cho, Fuchu-shi, Tokyo 183-8509, Japan

## Abstract

Enalapril maleate, the oldest and most widely distributed ACEI, and alacepril, the newest and antioxidant ACEI, were compared in the point of cardioprotective effect for Dahl salt-sensitive rat. In order to evaluate the correlation between the three factors, cardiac fibrosis and blood pressure/oxidative-stress marker (tissue TBARS), index of correlation was calculated. The results showed a significant difference in cardiac fibrosis between high-dose alacepril (30 mg/kg/day, group H) and enalapril maleate (10 mg/kg/day, group E). There was significant correlation between cardiac fibrosis and oxidative-stress marker, although there was no correlation between cardiac fibrosis and blood pressure. Fibrosis was more influenced by oxidative stress not by blood pressure, we should not select ACEI only by blood pressure-lowering effect and should more consider cardioprotective effects of ACEI.

## 1. Introduction

 Heart failure is defined as a syndrome that demonstrates a systolic dysfunction, diastolic dysfunction, or both. There are many mechanisms by which heart failure can be induced. However, cardiac remodeling and sequential fibrosis of cardiac muscle seems to contribute equally to cardiac failure. Although many factors are referred to as causative agents in cardiac remodeling, the renin-angiotensin system (RAS), sympathetic nerve system, and reactive oxygen species (ROS) are considered the major factors that intricately interact with each another. Because angiotensin II (Ang II) is generated by the RAS and induces the activation of sympathetic nerve system and augmentation of ROS, regulation of the RAS is recognized as an important measure for preventing fibrosis of cardiac muscle.

 Two types of RAS, circulating RAS and cardiac RAS, which act in different ways have been reported [1–6]. Circulating RAS is activated in response to decreased cardiac output where it increases blood pressure by constricting the peripheral vessels mainly via sodium retention. Cardiac RAS directly stimulates cardiomyocyte hypertrophy and fibroblast proliferation, which leads to left ventricular hypertrophy, diastolic and systolic dysfunction, arrhythmia, heart failure, and, ultimately, cardiac cell death. To maintain the cardiac function, inhibition of cardiac RAS seems important, and many types of angiotensin-converting enzyme inhibitors (ACEIs) are used for this purpose [7].

 Various types of ACEIs with very similar basic vasodepressive effects are available; however, they differ slightly in their pharmacokinetics. To stabilize the therapeutic effect and minimize the adverse effects, the currently available ACEIs are in the form of prodrugs and need to be converted into an active form in the body before they take effect. In addition, some ACEIs may act more directly on ACE in tissues than on ACE in the circulating blood [8]. Currently, five such ACEIs are commercially available for veterinary use; however, these drugs have not been fully investigated through comparative studies to determine which of these drugs is more effective and advantageous in treating heart failure. For example, although alacepril is a long-acting, sulfhydryl-containing ACEI, very little information is available regarding its cardioprotective effects [9]. In the present study, we evaluated the cardioprotective effects of alacepril and compared the effects with another ACEI, enalapril, which is widely used in veterinary medicine.

## 2. Materials and Methods

The experimental procedures were approved by the Institutional Animal Care and Use Committee of the Tokyo University of Agriculture and Technology. Three-week-old Dahl salt-sensitive rats (DS rats; Saitama Experimental Animals Supply, Saitama, Japan) were fed a low-salt (0.3% NaCl) diet (BP 168.8 ± 52 mmHg), and by 7 weeks of age, they were switched to a high-salt (8% NaCl) diet. They were then divided into four subgroups that received a low dose of alacepril (10 mg/kg/day, Group L), a high dose of alacepril (30 mg/kg/day, Group H), enalapril (10 mg/kg/day, Group E), and placebo (5% gum Arabic solution, Group P). From 7 weeks of age, noninvasive blood pressure measurements were obtained every 2 weeks using a tail-cuff method (MK-2000, Muromachi Kikai Co., Ltd., Tokyo, Japan). Echocardiographic studies were performed using a phased array sector scanner (SSD-5000, Aloka, Tokyo, Japan). Under general anesthesia with intraperitoneal administration of ketamine hydrochloride (60 mg/kg) and xylazine hydrochloride (5 mg/kg), a right parasternal short-axis image was obtained. In the papillary muscles, the size of the diastolic thickness of the interventricular septum (IVSd, mm), the diastolic thickness of the interventricular septum (IVSs, mm)), the diastolic thickness of the left-ventricular posterior wall (LVPWd, mm), the systolic thickness of the left-ventricular posterior wall (LVPWs, mm), the left-ventricular internal dimension at end diastole (LVIDd, mm), and the left-ventricular internal dimension at end systole (LVIDs, mm) were measured with M-mode measurement, and the fractional shortening (%FS) was calculated from LVIDs and LVIDd. A left parasternal apical four-chamber view was then obtained to record mitral inflow. The E and A peaks were measured, and the A/E ratio was calculated. Each echocardiographic measurement was repeated three times, and their average value was used for assessment.

 At 19 weeks of age, the rats received a lethal injection of sodium pentobarbital. Blood samples were collected from the vena cava, after which a midline thoracotomy was performed and the heart was removed and cut cross-sectionally at the papillary muscle. The apical region of the heart was used for the assay of oxidative stress, and the basal region for the measurement of cardiac fibrosis. Both blood and cardiac samples were immediately frozen and stored at −80°C until the measurements were obtained.

Urine 8-hydroxy-2′deoxyguanosine (8-OHdG) and plasma and tissue thiobarbituric acid reactive substances (TBARS) were used as indexes of lipid peroxidation and oxidative stress. 8-OHdG levels were determined by the method previously reported using commercial assay kit (Highly sensitive ELISA kit for 8-OHdG, JaICA, Hukuroi, Japan), applicable to samples from human, mouse, rat, rabbit, dog, cattle, and horse [10]. After incubation and washing, an anti-mouse secondary antibody conjugated to horseradish peroxidase was used to determine the bound monoclonal antibody. The color yield was directly proportional to the analyte concentration. Data reduction was conducted by four-parameter logistic curve fit. TBARS levels were determined by the method previously reported using commercial assay kit (TBARS Assay Kit, Cayman Chemical Company, Michigan, USA) [11]. Briefly, serum samples and homogenized tissue samples were incubated with TBA-working solution and the colored complex was determined at 532 nm, and TBARS concentration was then calculated using a calibration curve constructed from 1,1,3,3 tetraethoxypropane. Urine 8-OHdG and plasma/tissue TBARS were expressed in concentrations (ng/mL, nmol/mL and nmol/mg protein, resp.).

 Left ventricular histological examination was conducted to quantify the cardiac fibrosis. The ventricles were paraffin-embedded and cut into thin sections (thickness: 3 *μ*m) in the usual manner. They were then stained with hematoxylin-eosin and picrosirius red stain. The collagen density percentage was determined in the left ventricle. Each region was assessed following staining with picrosirius red in 10 random fields with a magnification of ×200. The collagen density percentage was calculated using a computerized morphometry system (Mac Scope Ver 2.69.1, Mitani Co., Fukui, Japan), and the sum of all the areas stained positive for sirius red was divided by the sum of all myocardial areas for each individual rat.

Statistical analysis was conducted using commercial software (PRISM 5 for Mac, GraphPad software Inc., CA, USA). Results are expressed as mean ± SD. Differences at specific stages between groups were assessed using one-way ANOVA and Bonferroni's multiple comparison test. A probability value of *P* < 0.05 was considered statistically significant.

## 3. Results

**Figure 1:**
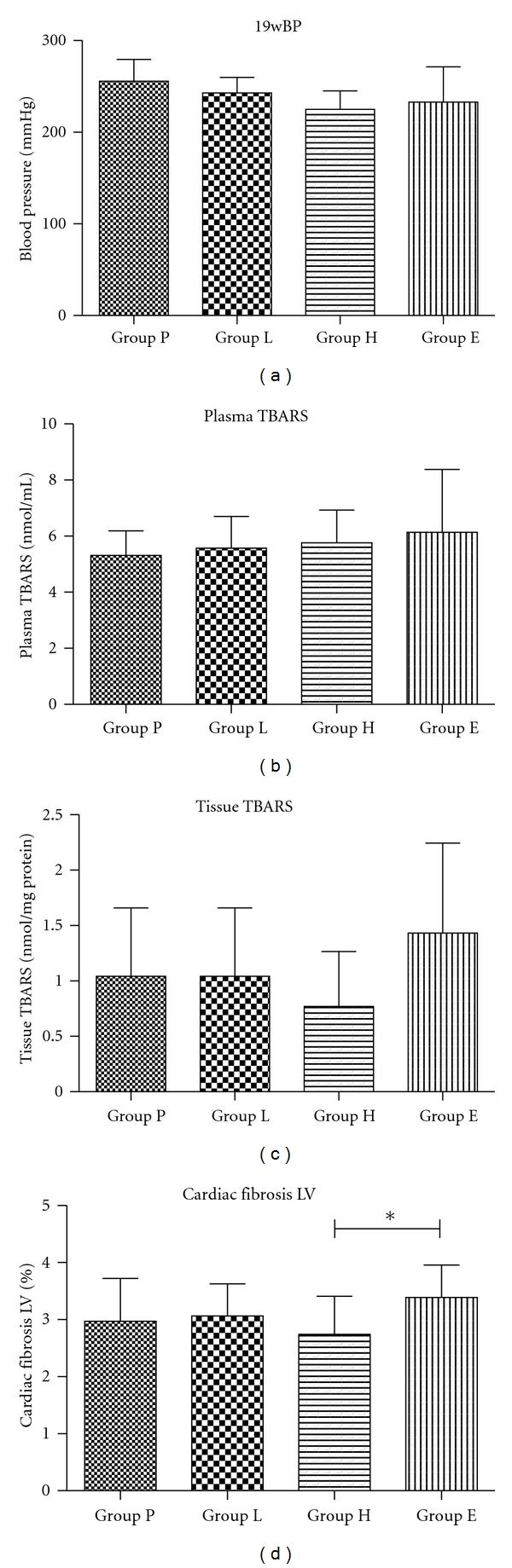
The mean blood pressure (mmHg), plasma TBARS (nmol/mL), tissue TBARS (nmol/mg protein), and cardiac fibrosis in LV (%) after ACEI administration. Cardiac fibrosis in LV was decreased in Group H and increased in Group E, so that there was a significant difference between Group H and Group E.

 A total of 40 DS rats (10 rats in each group) were used in the present study. The mean blood pressure before ACEI administration was 136.9 ± 4.2 mmHg, and a significant increase were observed in each group at 19 weeks of age (Group P: 255.6 ± 23.9 mmHg; Group L: 242.8 ± 17.2 mmHg; Group H: 224.9 ± 20.2 mmHg; Group E: 236.9 ± 38.4 mmHg, Figure 1). Group H showed suppressive effect, although there was no significant difference between the groups. There was no significant difference in the heart rates between the groups.

 Detailed data from the echocardiographic studies are summarized in Table 1. At 19 weeks of age, significant decreases in IVSd, IVSs, and LVPWd were noted in Group E; however, there were no significant changes in these findings in Groups L, H, and P. In addition, no statistically significant difference was observed among the ACEI administration groups (Groups L, H, and E). Also, no significant difference in %FS was noted before and after ACEI administration.

 The detailed data relating to urine 8-OHdG, plasma, and tissue TBARS and fibrosis are summarized in Table 2. There were no differences between the groups except for cardiac fibrosis. On left ventricular histological examination, cardiac fibrosis was lowest in Group H (2.74 ± 0.66%), and there were significant differences between Group E (*P* = 0.014). There were no significant differences between Group E and Group P.

**Figure 2:**
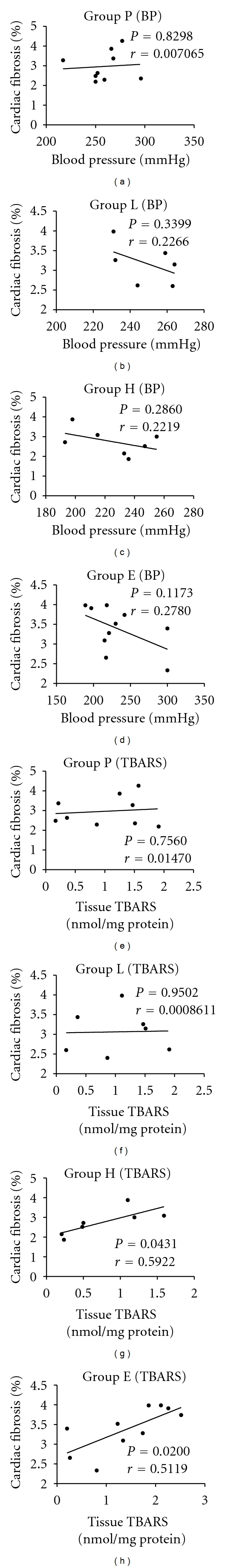
The coefficient of correlation between cardiac fibrosis and blood pressure (mmHg)/tissue TBARS (nmol/mg protein). Tissue TBARS in Groups H and E were significantly correlated with cardiac fibrosis in the left ventricle, whereas there was no significant correlation between cardiac fibrosis and blood pressure.

 The coefficient of correlation between cardiac fibrosis and blood pressure (mmHg)/tissue TBARS (nmol/mg protein) is shown in Figure 2. Tissue TBARS in Groups P and L were not significantly correlated with cardiac fibrosis in the left ventricle and blood pressure (*P* = 0.76 and *P* = 0.95, *r* = 0.01 and *r* = 0.001, resp., for cardiac fibrosis and *P* = 0.92 and *P* = 0.52, *r* = 0.001 and *r* = 0.07, resp., for blood pressure). Tissue TBARS in Groups H and E were significantly correlated with cardiac fibrosis in the left ventricle (*P* = 0.04 and *P* = 0.02, *r* = 0.59, and *r* = 0.51, resp.), whereas there was no significant correlation between cardiac fibrosis and blood pressure (*P* = 0.72 and *P* = 0.09, *r* = 0.02, and *r* = 0.31, resp.).

## 4. Discussion

DS rat have been reported to show steep elevation in blood pressure depending on the amount of salt intake [12, 13]. Elevation in blood pressure and progressive left ventricular hypertrophy have been observed at 4 to 5 weeks, resulting in overt diastolic heart failure at approximately 19 weeks [14]. In the present study, ACEI was administered from weeks 7 to 19, a timeframe that was anticipated to encompass the early stage through the end stage of hypertensive heart failure. However, high blood pressure occurred by high-salt food showed large variation and there are some difficulty in comparing drug effect between each group.

No differences in blood pressure were observed between the groups. At 19 weeks of age, ACEI administration began to decrease the blood pressure, especially in Group H. The decrease in blood pressure observed in the high-dose alacepril group could have been due to the drug potency; alternately, an antiadrenergic or antioxidant effect, which only alacepril possesses, might have been acting synergistically to promote the hypotensive effect.

To study the antiadrenergic effect of alacepril, cardiac rate was monitored. There were no significant differences between groups, and a direct antiadrenergic effect of alacepril was not confirmed. The cardiac rate of the conscious DS rats is about 500 beats/min, and this rate might have been elevated because of the stress of the blood pressure measurement in the present study. Assessment of cardiac rate therefore seems unsatisfactory as a method of estimating the antiadrenergic effect; another method, such as measurement of blood norepinephrine concentration, might provide a more accurate alternative.

Antioxidant effect was estimated by three parameters: 8-OHdG in blood plasma and urine, and tissue TBARS in heart muscle. 8-OHdG is released into cytoplasm when deoxyguanine is attacked by oxidative damage. Released 8-OHdG is not metabolized; it circulates in the blood and is then excreted as a filtrate in the urine [15]. Because 8-OHdG is easily measured in blood plasma and urine, its presence was expected to be a marker of oxidative stress available for repeat measurement. However, 8-OHdG showed no differences between the groups. In fact, 8-OHdG has dynamic physiological variation, especially in urine, and does not reflect the condition of the heart. Therefore, it cannot be considered as an index for determining the cardiac effects of ACEIs. On the other hand, tissue TBARS in Group H showed a significant decrease compared with Group E. TBARS are generated when the phospholipids in the cell membrane are oxidized by lipoperoxide; therefore, it is used as a parameter of oxidative damage in cell membrane [16]. However, TBARS also can be generated in other ways. For accurate measurement, we should measure samples on a like-for-like basis and compare the results in relative ways [17]. Because the samples for TBARS in the present study were limited to tissues obtained from the heart base of DS rats, and the sample collection and measurement were conducted using the same conditions, the results of TBARS indicated the relative level of free radicals. The decrease in free radicals observed in Group H compared with Group E was caused by the antioxidant effect of the sulfhydryl group present in desacetylalacepril and captopril, along with the pressure load reduction, which contributed to the reduction in oxidant stress. Through these mechanisms, high dose of alacepril could reduce the production of ROS in cardiac muscle tissue and provide a cardioprotective effect.

 Measurement of cardiac fibrosis in the left ventricle represents the amount of cardiac remodeling of the heart exposed to high blood pressure for 12 weeks. Although decreased ROS levels were observed in Group H, no significant decrease in cardiac fibrosis nor blood pressure was observed between Group P. One reason for this result was that Group P showed high dispersion of the drug, which might have been related to dispersion in salt-sensibility, pressure overload, and reactivity to the ROS. In addition, the rate of cardiac fibrosis was low in each group, and therefore it was not considered as a significant difference except for between Group H and Group E. From our previous study, we had thought that 12 weeks would be sufficient to induce cardiac failure; however, a longer observation period might be necessary [18]. Cardiac fibrosis in the left ventricle showed significant differences between Groups H and E. However, there were no significant differences against Group P, and therefore we were unable to determine whether the result was derived from the increased fibrosis in Group E or from the decreased fibrosis in Group H. To interrelate cardiac fibrosis with other factors at an individual level, the coefficient of correlation against blood pressure and tissue TBARS was calculated. Strong correlations were noted between cardiac fibrosis and tissue TBARS in Groups E and H; however, there were no significant correlations between cardiac fibrosis and blood pressure or between cardiac fibrosis and tissue TBARS in Groups P and L. Because cardiac fibrosis showed no significant negative correlation with blood pressure, there were assumed to be no causal association between cardiac fibrosis and blood pressure in the spontaneously hypertensive rats. The correlation between cardiac fibrosis and tissue TBARS showed large variations between the groups. Oxidative stress has been implicated in the pathogenesis of Ang-II-related hypertension and regulates the renewal or senescence of stem and progenitor cells [19]. Because TBARS can also be inhibited by ACEIs or angiotensin receptor blockers through the antiangiotensin II mechanism independently of blood pressure reduction [20–22], the anti-oxidative mechanism of the sulfhydryl group possessed by desacetylalacepril was very difficult to estimate. Although more detailed studies will be needed to clarify the precise antifibrotic mechanism of alacepril, it was suggested that cardiac fibrosis was influenced more strongly by ROS than by blood pressure under ACEI administration.

 Cardiac hyperplasia was observed at the posterior wall of the left ventricle and the interventricular septum on echocardiography at 19 weeks of age. Pressure overload was the main reason for the hyperplasia, and ACEIs showed a suppressive effect on its development. Significant differences were observed between Groups P and E, but not between the groups given ACEIs. It has been reported that the rennin system is not affected by salt-induced hypertension in the DS rat and the sympathetic nervous system is more involved in this model [23]. Systolic function was maintained through the drug administration schedule because the %FS did not alter during this period. Based on the findings relating to cardiac fibrosis, no remodeling appears to have occurred, which causes serious systolic dysfunction. Because the posterior wall of the left ventricle of the DS rat is only 2 mm thick and the heart rate was as rapid as 200 beats/min, it was difficult to judge the extent of cardiac fibrosis from the results of echocardiography alone.

## 5. Conclusions

 The present study reported relatively low levels of cardiac fibrosis in DS rats used in the present study, and this study model seemed to be inappropriate for the evaluation of ACEI for suppression of fibrosis. However, individual analysis of the rate of fibrosis, ROS, and blood pressure enabled us to demonstrate the relationship between these factors. The results showed the importance of ROS in heart muscle, and suggest that we should pay more attention to this factor when we use ACEIs to treat patients with heart failure. In the present study, we used the pressure overload model to evaluate the direct sympathoinhibitory action and free radical scavenging effect more clearly; however, the general indication for use of alacepril in veterinary medicine is volume overload such as mitral regurgitation. We should also evaluate the effect of alacepril using a volume overload model from the perspective of its free radical scavenging effect.

## Figures and Tables

**Table 1:** Echocardiographic data in 19 weeks. Detailed data from the echocardiographic studies. No statistically significant difference was observed among the ACEI administration groups in IVSd, IVSs, LVPWd, and %FS.

	IVSs (mm)	LVIDs (mm)	LVPWs (mm)	IVSd (mm)	LVIDd (mm)	LVPWd (mm)	FS (%)	E/A
Group P	0.73 ± 0.07	0.48 ± 0.07	0.31 ± 0.02	0.28 ± 0.03	0.48 ± 0.07	0.26 ± 0.01	33.6 ± 4.2	0.84 ± 0.13
Group L	0.76 ± 0.10	0.53 ± 0.05	0.27 ± 0.05	0.25 ± 0.05	0.53 ± 0.09	0.25 ± 0.04	29.6 ± 4.9	0.68 ± 0.07
Group H	0.83 ± 0.10	0.54 ± 0.05	0.28 ± 0.04	0.24 ± 0.03	0.54 ± 0.11	0.23 ± 0.03	35.1 ± 7.1	0.83 ± 0.18
Group E	0.82 ± 0.04*	0.55 ± 0.03	0.27 ± 0.02	0.24 ± 0.02*	0.55 ± 0.09	0.22 ± 0.03*	32.8 ± 8.4	0.66 ± 0.35

Mean ± SD, *means significant decrease compared with Group P.

**Table 2:** Urine 8-OHdG, plasma, tissue TBARS, and fibrosis. The detailed data relating to urine 8-OHdG, plasma, and tissue TBARS and fibrosis. Cardiac fibrosis was lowest in Group H, and there were significant differences between Group E (*P* = 0.014).

	Plasma TBARS (nmol/mL)	Urine 8-OhdG (ng/mL)	Tissue TBARS (value per protein, nmol/mg protein)	Tissue TBARS (value per weight of tissue, nmol/g)	Cardiac fibrosis (%)
Group P	5.6 ± 2.5	5.3 ± 0.9	1.0 ± 0.6	61.5 ± 35.3	2.971 ± 0.7517
Group L	6.5 ± 4.0	5.6 ± 1.1	1.4 ± 0.5	82.1 ± 30.5	3.065 ± 0.5602
Group H	7.2 ± 3.5	5.8 ± 1.2	0.8 ± 0.5	46.4 ± 30.2	2.744 ± 0.6649*
Group E	6.6 ± 3.0	6.2 ± 2.2	1.3 ± 0.8	80.9 ± 48.1	3.390 ± 0.5650

Mean ± SD, *means significant decrease compared with Group E.

## Abbreviations

ACEI: Angiotensin-converting enzyme inhibitors
RAS: Renin-angiotensin system
ROS: Reactive oxygen species
8-OHdG: 8-hydroxy-2′deoxyguanosine
TBARS: Thiobarbituric acid reactive substances
IVSd, mm: The diastolic thickness of the interventricular septum
IVSs, mm: The systolic thickness of the interventricular septum
LVPWd, mm: The diastolic thickness of the left-ventricular posterior wall
LVPWs, mm: The systolic thickness of the left-ventricular posterior wall
LVIDd, mm: The left-ventricular internal dimension at end diastole
LVIDs, mm: The left-ventricular internal dimension at end systole
%FS: The fractional shortening.
